# Computed tomography provides a “one-stop-shop” targeted analysis for coronary artery calcification and osteoporosis: a review

**DOI:** 10.3389/fendo.2025.1356831

**Published:** 2025-02-28

**Authors:** Jing Luo, Qian Wang, Wenhong Liu, Huazhi Liao, Weipeng Qing, Minyi Zhang, Deqiu Tang, Guanghua Luo, Heng Zhao

**Affiliations:** ^1^ Department of Radiology, The First Affiliated Hospital of University of South China, Hengyang, Hunan, China; ^2^ Department of Radiology, Hong’an County People’s Hospital, Huanggang, Hubei, China; ^3^ Major in Medical Imaging, The University of South China, Hengyang, Hunan, China

**Keywords:** computed tomography, coronary artery calcification, osteoporosis, bone-vascular axis, “one-stop-shop” analysis

## Abstract

The global trend towards longer lifespans has led to an aging population and a rise in the prevalence of diseases that predominantly affect elderly people. Coronary artery calcification (CAC) and osteoporosis (OP) are common in elderly populations. CT scans provide a reliable method to assess and monitor the progression of these diseases. In this review, the relationship between OP and CAC in terms of pathophysiological mechanism, comorbidity risk factors and clinical manifestations is reviewed, with a focus on the advancements in CT imaging, clinical applications and the possibility for “one-stop-shop” for examination.

## 1 Introduction

The global trend towards longer lifespans has led to an aging population and a rise in the prevalence of diseases that predominantly affect elderly people (1). Coronary artery disease (CAD) and osteoporosis (OP) are two clinical diseases influenced by aging, and often coexisting (2). Coronary artery calcification (CAC) is a marker of coronary atherosclerosis, indicating the presence of CAD, and regardless of the presence of risk factors or symptoms, serving as a definite predictor of future cardiac events (3, 4). Cardiovascular calcification is a widespread condition that that primarily affects the elderly population. Surprisingly, it is even more prevalent in OP patients (5). Compared with the general population, individuals with reduced bone mass and OP are at a higher risk of cardiovascular diseases. In one study, Chen et al. found that OP patients had a higher risk of CAD (6). Likewise in the presence of CAD, patients with low bone mineral density may have higher incidence rate and mortality, irrespective of age or conventional cardiovascular risk factors (2). Vascular calcification (VC) is an active process similar to bone formation and it is controlled by complex enzymatic and cellular pathways (7, 8). OP and cardiovascular calcification share common risk factors, including age, sex, inflammation and unhealthy lifestyle, which may partly explain the association between OP and CAC (9). Both diseases can be prevented or slowed by alleviating or treating modifiable risk factors. With the aging of global population and the rapidly growing burden of complications, the clinical applications of computed tomography (CT) scans continues to grow. Despite this, OP remains largely underdiagnosed and undertreated, leveraging CT images acquired for diverse indications (e.g., chest, heart, coronary artery, etc.) to opportunistically screen for OP offers a promising strategy to enhance its detection rate. CAC can be easily quantified via CT and expressed as a CAC score (CACS). This means that a single CT scan could potentially be used to evaluate both OP and CAC.

In this review, the relationship between OP and CAC in terms of pathophysiological mechanism, comorbidity risk factors and clinical manifestations is reviewed, with a focus on the advancements in CT imaging, clinical applications and the possibility for “one-stop-shop” examination.

## 2 The correlations between osteoporosis and coronary artery calcification

### 2.1 Clinical manifestations

There is a growing body of studies regarding the correlation between CAC and BMD, however, there are still conflicting results regarding these two age-related disorders. A study conducted in the Copenhagen general population reported a negative correlation between BMD and CAC in both men and postmenopausal women. This suggests that reduced bone density may increase the risk of CAC, regardless of gender. Xu et al. investigated the relationship between BMD and CAC in postmenopausal women, finding that women with low BMD were at a higher risk for CAC (10). In contrast, a study from China found no direct relationship between OP and CAC in elderly men after adjusting for age and other influencing factors (11). A study in middle-aged women had shown a direct association between high CAC and BMD, but no association in adjusted group for age and shared risk factors (12). Additionally, BMD varies by anatomical site and the relationship between BMD at different measurement sites and CAC also differs. A study on postmenopausal women also suggested that BMD at the spine and femoral neck, measured by dual-energy X-ray absorptiometry (DXA), were independent markers for an increased risk of CAC (10). Similarly, a Swedish study reported that the BMD in the trabecular bone volume of the 12th thoracic vertebra vertebra, measured by quantitative computed tomography (QCT), was negatively correlated with CAC in women, but the BMD in the cortical bone volume of the right femur was directly and independently correlated with CAC (13). This opposite relationship may be explained by the differences in mineral regulation of trabecular bone and cortical bone and may indicate distinct pathophysiologic mechanisms exist for the trabecular vs cortical bone in the bone-vascular axis (**Table 1**).

**Table 1:** Associations of BMD and CAC.

Study	References	BMD analysis tool	Locations	Groups	Simple size	Gender	Age (Mean±SD) or (Median(IQR))	Prevalence of CAC	BMD values (Mean±SD)(mg/cm^3^) or (and) T score		Results
Ghada N Farhat et al.	(12)	QCT	Lumbar vertebrae	No CAC	490	Female(490)	49.6 ± 2.7	47.76%	163.8 ± 37.1	−0.18 ± 1.43	Direct association between high CAC and vBMD in unadjusted but not adjusted models; No associations of between moderate CAC and vBMD
				Moderate CAC (0-7.54)			50.2 ± 3.1		166.1 ± 37.3	−0.09 ± 1.43	
				High CAC			50.9 ± 2.6		153.1 ± 36.4^#**^	−0.59 ± 1.4^#**^	
Joseph A.Hyder et al.	(14)	QCT	Lumbar vertebrae	NA	1909	Female(946)	65.2 ± 9.2	47.00%	111 ± 13		Inverse association between CAC and BMD in women, but not in men
						Male(963)	64.1 ± 9.9	68.00%	121 ± 39		
Jimmy J Chan et al.	(15)	QCT	Lumbar vertebrae	Categorized by sex-specific quartiles (Q4=high vBMD)	1317	Female(689)	60 ± 9	57.00%	NA		Inverse association between CAC and integral vBMD, trabecular vBMD; No association between CAC and cortical vBMD in women
						Male(628)	60 ± 9	86.00%			No association between CAC and integral vBMD,trabecular vBMD, and cortical vBMD in men
Xu Rui et al.	(10)	DXA	Left femoral neck	CACS=0	186	Female(186)	63.8 ± 6.9	58.60%	L.aBMD (-0.87 ± 1.03)	F.aBMD(− 0.76 ± 0.98)	Inverse association between CAC and lumbar aBMD, femoral neck aBMD
			Lumbar vertebrae	CACS>0			66.8 ± 7.4		L.aBMD (−1.36 ± 1.42)^#**^	F.aBMD(− 1.18 ± 1.23)^#***^	
N. Ahmadi et al.	(16)	QCT	Thoracic vertebrae	CACS=0	5590	Female(2017)	51 ± 11	58.70%	178.4 ± 10.4		Inverse association between CAC and BMD in both women and men
				CACS 1-100			57 ± 10		169.6 ± 20.3		
				CACS 100-400		Male(3573)	62 ± 10		155.3 ± 21.4		
				CACS–400			66 ± 10		145.2 ± 21.6^#***^		
Yaffah L. Wiegandt et al.	(17)	QCT	Thoracic vertebrae	NA	2366	Female(1322)	60 ± 10	41.00%	151 ± 49		Inverse association between CAC and BMD in both men and postmenopausal women
						Male(1044)	61 ± 11	69.00%	138 ± 46		
Funck-Brentano T et al.	(13)	QCT	Thoracic vertebra	CACS=0	1060	Female(519)	56.9 ± 4.2	NA	Tb.vBMD(114 ± 26)	Ct.vBMD(926 ± 37)	In women, inverse association between CAC and trabecular vBMD; Direct independent association between CAC and cortical vBMD
							59.4 ± 4.1^***^		Tb.vBMD(107 ± 23)^#**^	Ct.vBMD(938 ± 35)^#**^	
			Right midshaft femur	CACS>0		Male(541)	56.5 ± 4.4		Tb.vBMD(116 ± 26)	Ct.vBMD(928 ± 38)	In men, no association between CAC and trabecular vBMD, cortical vBMD
							58.6 ± 4.3^***^		Tb.vBMD(114 ± 26)	Ct.vBMD(932 ± 39)	

# indicates statistical differences between groups; *(p<0.05),**(p<0.01),***(p<0.001).

### 2.2 Mechanism

**Figure 1:**
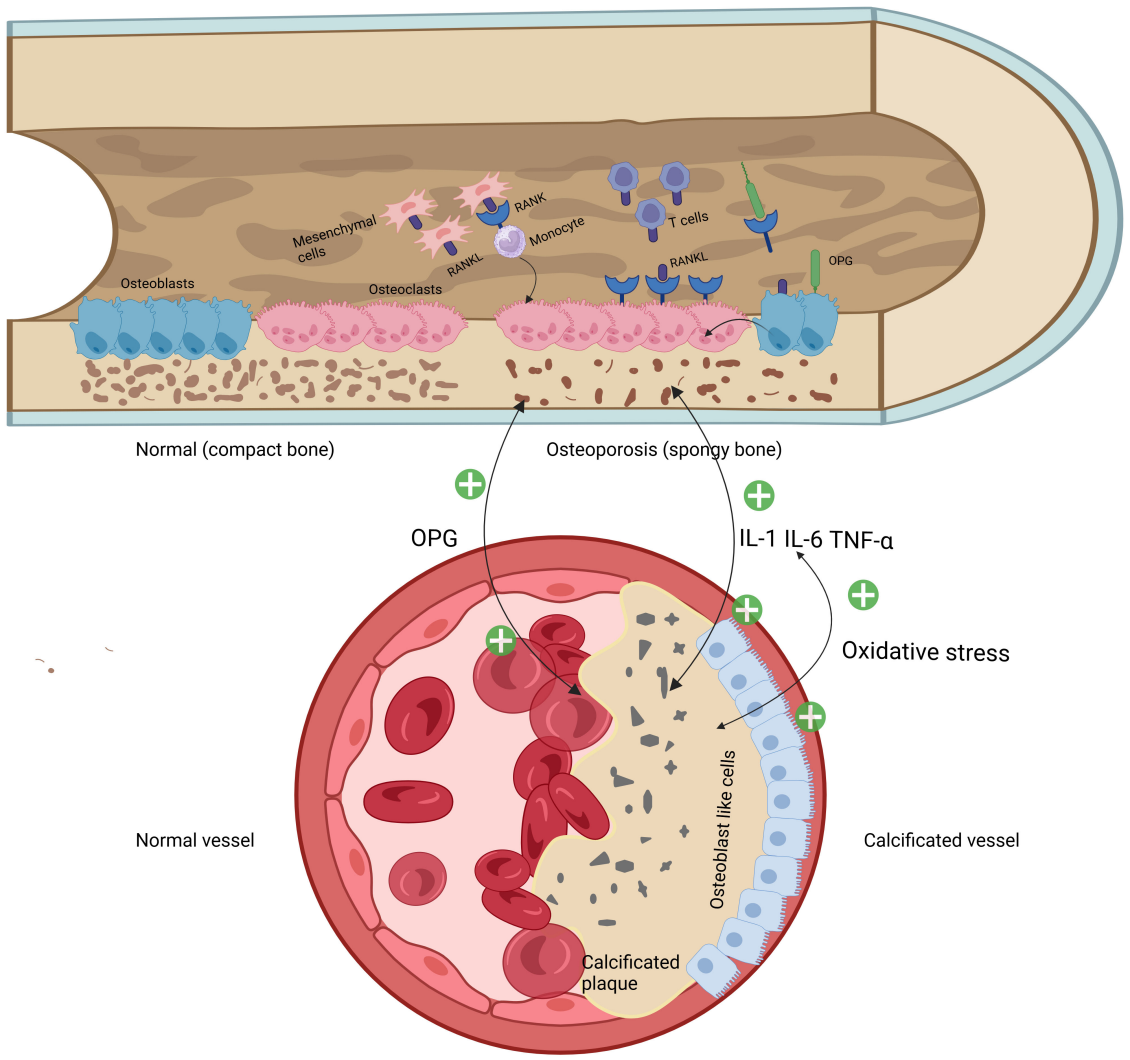
Mechanical diagram of bone-vascular axis regulation.

Biomineralization is considered as the deposition of minerals in an organized manner on a matrix (18). Apart from the skeleton, the most common structure in humans to undergo calcification is the vascular system, where VC represents an active regulatory form of tissue biomineralization (19). Mineralization is crucial for the maintenance of normal skeletal health. However, the deposition of minerals in soft tissues, especially in the vascular system, is typically pathological and linked to an increased risk of adverse cardiovascular events (20). Notably, ectopic arterial calcification is frequently accompanied by decreased BMD or disturbed of bone turnover (21). At the molecular level, the various biomarkers involved in the osteogenic processes can be discovered in calcified vascular segments (22). Cellular, endocrine and metabolic signals flow bidirectionally between the vascular system and the skeleton, which is essential for the maintenance of both bone and vascular health (19) (**Figure 1**).

#### 2.2.1 Cellular pathways

Osteogenesis is a process carried out in two steps: the secretion of extracellular matrix by osteoblasts and the subsequent mineralization of the matrix (23). This process is regulated by numerous cellular pathways and bone-related proteins, such as the RANK/RANKL/OPG pathway, osteopontin (OPN), bone morphogenetic proteins (BMP) and alkaline phosphatase (ALP). Among these, the RANK/RANKL/OPG pathway plays a critical role in maintaining bone homeostasis and is also implicated in various pathological processes, including atherosclerosis and cardiovascular diseases (24).

RANKL is a type II transmembrane protein expressed by osteoblasts, osteocytes and immune cells within bone tissues. Its receptor, RANK is a type I transmembrane protein located on osteoclast progenitor cells, mature osteoclasts and immune cells (25). Under normal physiological conditions, the interaction between RANKL and RANK plays a pivotal role in stimulating osteoclastogenesis, bone remodeling, and maintaining calcium homeostasis (24). Osteoprotegerin (OPG), a member of the TNF receptor superfamily, is secreted by osteoblasts and acts by binding to RANKL and disrupting the RANK-RANKL interaction, inhibiting osteoclast formation and bone resorption (26). The RANK/RANKL/OPG pathway has emerged as a potential connection between osteoporosis and coronary artery CAC (27). Mice with OPG gene knockout exhibit both VC and brittle fractures (28).On the contrary, OPG treatment has been shown to prevent VC induced by vitamin D and warfarin in rats, as well as inhibit the calcification of vascular smooth muscle cells (VSMCs) *in vitro (* 29). These findings suggest that OPG may act as a molecular link between the vascular and bone systems. Paradoxically however, in human studies, serum OPG levels were increased in patients with CAD and correlated with their severity, ischemic cardiac decompensation, and future cardiovascular events (30, 31). They proposed that OPG may be passively elevated as a protective factor against vascular calcification. Therefore, it is possible that OPG could be a potential biomarker in early identification and monitoring patients with cardiovascular disease (32).

VC is an active regulatory form of extracellular matrix biomineralization. Ectopic biomineralization of the vascular walls begins with impaired endothelial function. Repeated endothelial dysfunction leads to excessive calcium transport from bones to the vascular walls, ultimately resulting in VC and OP (33). Osteopontin (OPN) is an extracellular matrix protein involved in bone metabolism and osteoclast activation. It facilitates the attachment of osteoclasts to the extracellular matrix by tightly binding to hydroxyapatite. Studies have confirmed that OPN is rarely expressed in normal arterial walls, but its expression is significantly elevated in the neointima formed after various pathological damages to the vessels, which proves that it can promote the adhesion, proliferation and migration of VSMCs and fibroblasts, and enhance the chemotaxis of macrophages (34). This eventually leads to the transformation of VSMCs from a contractile phenotype to an osteogenic phenotype. The discovery that bone morphogenetic protein (BMP) is abundantly expressed in calcified areas of atherosclerotic vessels preliminarily determines its ability to promote abnormal calcification (35–37). Subsequent research has shown that BMP ligands play a key role in the development and homeostasis of the vascular system (38). BMP-2 stands out as a cytokine with strong osteogenic activity and can induce ectopic osteogenesis (39). It can trigger cell chemotaxis, mitotic proliferation, cell differentiation and ultimately lead to ectopic bone formation, and the development of VC through mechanisms such as microRNAs, MGP, apoptosis, oxidative stress and hyperglycemia (40).

#### 2.2.2 Endocrine regulation

The endocrine system also participates in the regulation of calcification. An important process of VC is the transformation of VSMCs from a contractile phenotype to an osteoblastic phenotype. Among the molecules involved in this process, parathyroid hormone (PTH) plays a key role via multiple mechanisms, including the regulation of the RANK/RANKL/OPG system and the Wnt/β-catenin pathway, which are the main pathways for bone resorption and formation respectively (41). In addition, PTH also regulates the action of several VC promoters, like calcium, phosphorus and vitamin D.

Sex hormones, especially estrogen, regulate bone metabolism by acting on receptors on osteoblasts and osteoclasts, and a deficiency of sex hormones can eventually lead to OP (9). Estrogen reduces the responsiveness of osteoclast precursors to RANKL, interfering with osteoclast signaling pathways and inhibiting osteoclast differentiation. The withdrawal of estrogen after menopause affects the normal balance between RANKL and OPG, ultimately prolonging the life of osteoclasts and promoting bone resorption (42). Sex hormones are known to affect the development of atherosclerotic disease, with women experiencing a 10-15 year delay in the onset of the disease compared to men, possibly due to the protective effect of estrogen in the years prior to menopause. Increasing evidence has suggested that endogenous estrogen plays a protective role against CAD in postmenopausal women and its deprivation worsens CAD progression after menopause. The reduction in estrogen also increases the risk of OP (10, 43–45).

#### 2.2.3 Calcium-phosphorus metabolism

The mineral component of bone is hydroxyapatite (HA), with the chemical formula Ca10(PO4)6(OH)2, formed by inorganic phosphate and calcium ions (Ca2+). Calcium-phosphorus metabolism is regulated by a wide variety of local and systemic factors. The coordinated actions of these substances prevent both low and high mineralization, both of which are detrimental to bone function. For this reason, the balance between calcification inducers and calcification inhibitors in the body is crucial for maintaining biomineralization. Pyrophosphate (PPi) is a local inhibitor of mineralization. Extracellular PPi effectively inhibits the ability of Ca2+ to crystallize with Pi and form HA and it strongly binds to the surface of HAP, thereby preventing further crystal growth (46).

Alkaline phosphatase (ALP), a membrane-bound extracellular enzyme, facilitates calcification by hydrolyzing phosphodiester bonds to release phosphate (47). Tissue-nonspecific ALP (TNSALP)-knockout mice exhibit skeletal deformities, impaired bone mineralization, and premature mortality due to seizures before weaning, consistent with the vital role of ALP in bone health (48, 49). Research has shown that TNSALP expression increases in a calcification environment and this enzyme inhibition can reduce calcification in VSMCs (50, 51).

#### 2.2.4 Genetics

There exist some genes that contribute to the comorbidity of VC and OP. Previous study has shown that mice with OPG gene knockout exhibit VC and fragility fractures (28). As one of the OPG promoter polymorphisms (950 T→C), it is not related to OP, but is associated with vascular morphology and function (52). One study found that the proportion of OPG gene haplotypes significantly increased in CAD patients, and that serum OPG levels were also associated with the presence of the C allele at position 950, suggesting that variations in the OPG gene can increase the risk of CAD (53). The above researches indicate that defects in the OPG gene are closely related to the occurrence of OP and CAD. Moreover, a study indicated that biallelic ENPP1 deficiency can lead to systemic arterial calcification in infants, while a heterozygous ENPP1 deficiency may result in early-onset OP (54). This suggests that mutations in ENPP1 may also be a factor contributing to the co-morbidity of CAC and OP. However, there are currently few studies on the genetic links between CAC and osteoporosis, and further exploration is needed to determine whether there are other genes causing its changes.

## 3 Coexisting risk factors in OP and CAC

Both OP and CAC share certain risk factors, such as ageing, sex, inflammation and unhealthy lifestyle.

### 3.1 Ageing

Cardiovascular calcification is a common disease in the elderly population, especially affecting most people over the age of 60, and is more common in patients with OP (10). According to the latest statistics from the International Osteoporosis Foundation, in the global context, one-third of women over 50 and one-fifth of men will experience an osteoporotic fracture during their lifetime (17).

### 3.2 Sex

Lower BMD was associated with higher coronary calcification and coronary plaque burdens in postmenopausal women, independent of cardiovascular risk factors and age (55). Hyder et al. demonstrated an inverse association between CAC and lumbar BMD in women, but not in men (14). Similarly, Chan et al. displayed an inverse association between CAC and integral vBMD, trabecular vBMD in women, but not in men (15). This may be due to estrogen deficiency in postmenopausal women, which leads to decreased cardiovascular and bone protection.

### 3.3 Inflammation

Another potential mechanism linking CAC and OP is that both processes are tissue-specific responses to chronic inflammation. Calcification results from the impaired clearance of Ca2+ following tissue damage, particularly during inflammation (56). Within atherosclerosis, inflammatory processes are triggered by the accumulation and oxidative modification of lipids within the subendothelial region. Initial calcification manifests within the lipid-rich core of the atheroma, adjacent to infiltrating inflammatory cells within the fibrocalcific plaque (57, 58). Previous studies by Al-Aly et al (59) and Cheng (60) et al. have demonstrated that the inflammatory cytokine tumor necrosis factor-α exerts significant control over osteochondrogenic differentiation in osteoblastic and vascular cells via modulation of the Msx2 and Wnt signaling pathways.

### 3.4 Unhealthy lifestyle

Sedentary lifestyle is associated with both cardiovascular disease and osteoporosis (61).The health of bones depends on optimal nutrition. Fruits and vegetables contain many nutrients that are essential for maintaining bone health such as magnesium, vitamin C, potassium and carotenoids (62). Smoking has a direct impact on the physiological activity of nuclear factor kappa-B receptor activator (RANKL), osteoprotegerin and osteoblasts (63). Interestingly, quitting smoking has been reported to reverse its effects on BMD (64).

## 4 CT imaging in CAC

CAC is an independent risk factor of major adverse cardiovascular events (65). There are two main methods of CT to assess CAC, one is non-contrast CT to measure calcification score, and the other is coronary artery CT angiography (CCTA) to detect both calcified and non-calcified plaques and measure the resulting degree of luminal stenosis (66). CT is a non-invasive examination method commonly used in clinical practice, which with high sensitivity and specificity for detecting calcium, and is able to quantify calcification (67).

### 4.1 Non-contrast CT

Agatston et al. in 1990 initially described a quantitative index of CAC which was later named Agatston score. They acquired coronary artery images by an electrocardiogram (ECG)-gated non-contrast CT, 20 contiguous slices were obtained below the bifurcation of the main pulmonary artery, with a 100 ms scan time, a 3 mm slice thickness, each image slice was triggered at 80% of the patient’s RR interval. A calcific lesion is defined as lesion with a CT attenuation threshold of 130 Hounsfield units (HU) and an area greater than 1mm2. A calcification score for each lesion is calculated by multiplying the lesion area and the density score that is determined based on maximal CT attenuation of measured lesion (density score: 1 = 130 to 199, 2 = 200 to 299, 3 = 300 to 399 and 4 = ≥ 400), then add the score of calcified coronary lesions at all levels to calculate total Agatston score (68).

Agatston score remains the standard reference for quantifying CAC and most widely used in clinical practice (69). With the development of high-resolution CT, the coronary artery scanning protocol has also been optimized, and the parameters are as follows: 120 kVp with variable mA according to patient body weight, slice thickness of ≤1.25 mm can be obtained, scan range from the level of the tracheal carina to the bottom of the heart. CAC provides great value for risk stratification of patients with cardiovascular disease, and is the gold standard method of assessing subclinical atherosclerosis (70). A meta-analysis by Abuzaid et al. had showed that compared with absence of CAC, CAC > 0 was associated with an increased risk of major adverse cardiovascular and all-cause mortality in both asymptomatic and symptomatic population in patients without an established diagnosis of CAD (71). A large-scale study of multiple ethnicities with a median follow-up of 11.7 years showed that the risk of total and CAD mortality increased with increasing levels of CAC (72). Moreover, A diabetes heart study demonstrated that CAC score had a higher predictive value of cardiovascular risk than the Framingham risk score (FRS) (73). Therefore, CAC has an incremental predictive value, and CT can accurately quantify and grade CAC, which is of great guiding significance for clinical diagnosis and treatment. It is worth noting that multiple studies had shown patients with higher CAC had lower BMD (16, 74), and the prevalence of CAD increased in populations with OP or osteopenia (75). This reminds us the importance of simultaneously evaluating CAC and BMD to screen for osteoporosis in patients with CAD in clinical practice.

### 4.2 Coronary artery CT angiography

Compared to non-contrast CT, in addition to detecting calcified plaques, CCTA can also detect mixed plaques and non-calcified plaques, and identify vascular stenosis. CCTA is an excellent non-invasive imaging modality of excluding CAD (66). Although CAC was significantly associated with adverse cardiovascular events, no CAC did not completely rule out obstructive CAD (76), and minimal CAC is also not exactly an indication of a small risk of malignant cardiovascular events. Spotty calcification is a feature of vulnerable plaques (77). Studies have proposed that spotty calcification is more likely to develop into unstable plaques and rupture abruptly leading to myocardial infarction (MI), while extensive calcification tend to develop into stable plaques and cause myocardial ischemia and angina (78, 79). A prospective cohort study revealed that compared with individuals with the absence of CAC, those with a minimal CAC were more likely to have hypertension, dyslipidemia and a family history of heart attack (80). And people with minimal CAC had a threefold higher risk of developing coronary heart disease compared with a CAC = 0 (81). Moreover, a recent study showed that patients with CAC measured by CTA have lower bone mineral density compared with those without CAC in postmenopausal women, and every 10 units of CACS increase, the risk of OP increases by 2% (82). Therefore, it is important to rule out severe coronary artery disease and screen for osteoporosis even in populations with very low CACS.

However, it is worth noting that evaluating BMD through contrast-enhanced CT scans of patients can lead to severe overestimation, and in clinical practice, relevant adjustments need to be made before implementing contrast-enhanced imaging for BMD screening (83).

## 5 CT imaging in OP

OP remains largely underdiagnosed and undertreated. Clinically, BMD measured by DXA is commonly used to assess OP. Its advantages are being convenient to operate, low cost, and low radiation, while the disadvantage is that DXA can only measure area bone density, which cannot reflect the three-dimensional characteristics of the bone (84). Three-dimensional imaging by CT to assess OP can be a good way to overcome this disadvantage. Based on CT images clearly display the anatomical structure of bone, the BMD of trabecular, cortical, or integral bone, centrally or peripherally can all be quantitatively measured (85). BMD measurements by CT are less influenced by vascular calcifications, abnormal bone changes (e.g. spine degenerative diseases, scoliosis, kyphosis, etc.), patient positioning errors, and various artifacts (86). Quantitative computed tomography (QCT) is an excellent diagnostic tool for OP and equipped with those advantages.

### 5.1 Quantitative CT

As early as the 1980s, CT can be used to assess for OP (87). The measurement of BMD by QCT requires a special calibration phantom and professional post-processing software, on which the attenuation values on the clinical CT images can be converted to the equivalent density of hydroxyapatite (88). Compared with DXA, a projectional method for measuring BMD, QCT can measure cortical bone separately from trabecular bone, which has a larger surface area and is more metabolically active, allowing for more sensitive detection of OP (89). Traditional calibration phantoms are made with known amounts of HA or potassium phosphate and are placed under the subject’s lower back while simultaneously undergoing CT scanning, which referred to as “synchronous calibration” (86). However, calibration phantom is not convenient in clinical practice, because the vast majority of CT scans are not initially intended to assess for OP, thus the phantom is not scanned simultaneously with patients. Nowadays, there is an asynchronous calibration phantom can be scanned separately from the subject and then the CT images that have been scanned can be transferred by to specialized post-processing software for subsequent BMD assessment (90). Based on this asynchronous calibration, images taken for other purposes, such as chest, abdomen, pelvis, etc., can be retrospectively used to measure BMD, providing great convenience for clinical practice. Besides, Funck-Brentano T et al. demonstrated CAC was inversely associated with trabecular QCT volumetric BMD (QCT-vBMD) of thoracic vertebra, but was directly and independently associated with cortical QCT-vBMD of midshaft femur in women (13). This opposite connection may be due to differences in the regulation of trabecular and cortical bone, further reflecting differences in the mechanism of mineral transformation of coronary calcification to trabecular and cortical bone.

### 5.2 Peripheral QCT

Because of the spatial resolution of clinical whole-body CT scanners is not enough, measurements of cortical BMD in the femoral neck and the vertebral body may be insufficiently accurate (91). High-resolution peripheral QCT (HR-pQCT) scanners sufficiently improve spatial resolution for evaluating distal tibia and radius (91). It is an imaging technique designed for *in vivo* 3D evaluation of the volumetric BMD (vBMD), geometry and microstructure of distal radius and tibia, making it a unique tool for obtaining alternative quantitative measurements of bone strength. The small isotropic voxel size achieved by HR-pQCT (82mm or 61mm) can distinguish cortical and trabecular bone compartments, visualize and quantify trabecular microstructure and cortical porosity (92–94). Thus, additional information can be obtained for osteoporosis evaluation. A Framingham Study of QCT-vBMD and VC reported that CAC was negatively associated with integral vBMD, trabecular vBMD of lumbar vertebra, but no association with cortical vBMD (15). This may indicate that the mechanisms of cortical bone and trabecular bone in the bone-vascular axis are inconsistent. Despite this, due to the insufficient spatial resolution of clinical whole-body CT scanners, the measurement of vertebral and femoral cortical bones may be inaccurate. Therefore, HR-pQCT, specialized in the assessment of cortical bone, is necessitated to further evaluate the relationship between CAC and cortical bone BMD.

### 5.3 Routine CT

Although QCT is gradually replacing DXA as the gold standard for measuring BMD, it is still limited in clinical use due to the need for specific phantom and dedicated software for assessment of osteoporosis. Efforts are underway to validate the application of opportunistic screening based on clinical CT scans—i.e., without calibration phantoms—for assessing OP (95). While Josephine et al. measured pelvic bone density using two calibration techniques (phantom-based and non-phantom-based) and found insufficient consistency to support interchangeability (96). Nevertheless, multiple studies have shown that measuring trabecular HU in routine CT scans can be an effective tool for the opportunistic evaluation and screening of OP, yielding promising results (97, 98). The vast majority of CT images in clinical practice contain skeletal composition information, from which the HU values of trabecular bone can be conveniently obtained. Therefore, CT scans of the chest, abdomen, pelvis, and spine for any clinical indications can be used to screen for OP without additional burden to the patient of radiation exposure, examination time, or image acquisition cost (86), thus OP can be extensively screened and substantially improve diagnosis in clinical practice. There was a clinical studies shown that patients with lower thoracic BMD measured by cardiac CT have a higher risk of fractures (99). A large cohort study had reported that the threshold for distinguishing OP from osteopenia was 90% sensitivity at 160 HU and more than 90% sensitivity at 110 HU (98). A recent Chinese study had revealed an excellent correlation between the average volume HU values of L1 and L2 and BMD measured by QCT (r = 0.941, P < 0.001), and the optimal thresholds of ROC for diagnosing osteopenia and OP were 154.73HU (sensibility 92.9%) and 106.52HU (sensitivity 86.6%), respectively (100). These studies have drawn similar conclusions, confirming the effectiveness of volumetric HU in predicting the occurrence of OP. Therefore, HU value can be used as a supplement method for clinical screening of OP. Moreover, studies had shown a significant inverse association between the HU value of vertebrae and CAC (101, 102), and the CT values of thoracic cancellous bone were significantly negatively correlated with the number of branches involved in coronary artery calcification and severity, which synchronously obtained coronary calcification date and thoracic cancellous bone CT measurements in one-time coronary spiral CT scan, able to achieve “one-stop-shop” targeted analysis (101).

### 5.4 Dual-energy CT

As early as the 1980s, there were studies using dual-energy CT(DECT) to measure the bone mineral mass of human vertebrae *in vitro* (103, 104). Since different tissue components exhibit variable attenuation at two different rapid-switching X-ray spectral energy levels, DECT can distinguish different materials and identify diverse body contents (105, 106). Based on this material decomposition (MD) technology, DECT is superior to conventional single-energy CT in material separation, and it has been carried out in various musculoskeletal applications such as measuring bone density, analyzing bone composition, and detecting bone trauma, which provided more clinically relevant information (106–108). Studies had compared the accuracy of bone component values directly quantified by DECT MD technology with the vBMD values of vertebral bodies measured by QCT, and derived different results (109, 110). Zhou et al. further proposed an indirect quantification method through linear regression calibration, which demonstrated the indirect method was in good agreement with the QCT, while the direct method was not accurate enough (110), implying the MD technology does not directly reflect the content of human tissue. In this study, CT imaging was performed with a Revolution GSI CT scanner (GE Medical Systems, Milwaukee, WI, USA), then the raw image data were transmitted to an advanced workstation (ADW4.6; GE Medical Systems) – which is also equipped with an Agatston score analysis technique – for BMD assessment by MD technique, thus a single DECT scan can be used for simultaneous post-processing analysis of BMD and CAC.

## 6 “One-stop-shop” examination and prospects

With the continuous progress of mineral metabolism research *in vivo*, the view that OP and vascular calcification share common pathophysiology mechanism and interact with each other has been more and more recognized, and the bone-vascular axis has attracted increasing attention. Multiple studies had confirmed there were coexisting risk factors in both diseases, including aging, estrogen deficiency, inflammation, and unhealthy lifestyle (55, 111, 112), and there were complex relationships in age, sex and anatomical sites between these diseases (2, 56).

Many methods are able to visually and assess calcification *in vivo*. Due to its high contrast resolution and short examination time, CT is widely used in clinical practice and can be conveniently used to quantitative calcified components and effectively monitor their progression. Clinical whole-body CT scanners provide images for diagnostic purposes while also reflecting a variety of additional body composition information, such as the attenuation of calcification (or mineralization), with no additional increase in radiation exposure, examination time, or image acquisition costs (86). Therefore, A routine CT scan can simultaneously detect vascular calcification and OP and provide quantitative data. For example, coronary spiral CT image can be used to reflect the bone density of patients by simultaneously detecting the CT value of thoracic cancellous bone (101). Moreover, the images by routine CT involved coronary, heart or chest can be used to detect CAC and transferred to the QCT post-processing station for BMD measurement, thus we can achieve “one-stop-shop” examination for OP and CAC. In this way, the detection rate of OP may be substantially improved, the preventive measures can be actively taken in the primary care setting in patients with CAD.

## 7 Conclusion

Overall, numerous studies have shown that the bone-vascular axis is the common pathological basis for CAC and OP. The relationship between CAC and bone density varies across age, gender and different anatomical sites. CT provides a reliable means of synchronously evaluating CAC and vertebral BMD in single scan, and monitoring the progression and response to therapy. The “one-stop-shop” examination provides great help in the screening, prevention, and management of clinical diseases.
